# Body self-esteem is related to subjective well-being, severity of depressive symptoms, BMI, glycated hemoglobin levels, and diabetes-related distress in type 2 diabetes

**DOI:** 10.1371/journal.pone.0263766

**Published:** 2022-02-15

**Authors:** Andrzej Kokoszka, Agata Pacura, Barbara Kostecka, Cathy E. Lloyd, Norman Sartorius

**Affiliations:** 1 II Department of Psychiatry, Medical University of Warsaw, Warsaw, Poland; 2 SWPS University of Social Sciences and Humanities, Warsaw, Poland; 3 Faculty of Wellbeing, Education and Language Studies, The Open University, Milton Keynes, United Kingdom; 4 Association for the Improvement of Mental Health Programmes (AMH), Geneva, Switzerland; National Cheng Kung University College of Medicine, TAIWAN

## Abstract

**Background:**

There are limited data on the role of body image in patients with type 2 diabetes. The purpose of this study was to compare body self-esteem in this group with norms for the general Polish population and to investigate the relationship between body self-esteem and the psychological and clinical characteristics of the course of diabetes.

**Methods:**

A group of 100 consecutive adult patients with type 2 diabetes (49 women and 51 men) aged 35 to 66 years were assessed using the Body Esteem Scale (BES), World Health Organization-Five Well-Being Index (WHO-5), Problem Areas in Diabetes Scale (PAID), and Hamilton Rating Scale for Depression (HAM-D).

**Results:**

In comparison to norms for the general population, women with type 2 diabetes had lower body self-esteem only in the dimension of Physical Condition (M = 30.71; SD = 7.11 versus M = 32.96; SD = 5.69; *P* = 0.003), whereas men in the dimensions of Physical Condition (M = 42.43; SD = 9.43 versus M = 48.30; SD = 8.42; *P* <0.001) and Upper Body Strength (M = 32.16; SD = 6.60 versus M = 33.97; SD = 5.86; *P* = 0.015). There were moderate or weak positive correlations between the overall BES score and/or its dimensions and subjective well-being, and negative correlations between the overall BES score and/or its dimension and the severity of depression symptoms, level of glycated hemoglobin (HbA_1c_), body mass index (BMI), and diabetes-related distress among women. Among men, BES scores were positively correlated with well-being, and negatively, with BMI and diabetes-related distress. A correlation of *r* = 0.39 between BES scores and HbA_1c_ levels was relatively high compared with values for other psychosocial factors. Both in women and men, a high Physical Condition score was a significant predictor of better well-being, less severe depression, and milder diabetes-related distress. Among men, it was also a significant predictor of lower BMI, whereas among women, BMI was predicted by Weight Concern.

**Conclusions:**

Persons with diabetes seem to have lower body self-esteem than the general population, which is significantly associated with clinical and psychological characteristics of the diabetes course. The observed differences and relationships are gender-specific.

## Introduction

Achievement and maintenance of optimal blood glucose level in individuals with type 2 diabetes remains a challenge for modern medicine. The authors of the American Diabetes Association (ADA) position paper on psychosocial care for people with diabetes concluded that, in the light of the available research, management with a psychosocially sensitive treatment regimen improves the level of glycated hemoglobin (HbA_1c_), but this effect is negligible [1]. Conversely, some empirical studies have shown that improvement in psychological well-being may reduce the risk of disease complications and helps achieve better metabolic control (e.g. [2–4]). According to the current ADA treatment guidelines, “Psychosocial care should be integrated with a collaborative, patient-centered approach and provided to all people with diabetes, with the goals of optimizing health outcomes and health-related quality of life’” [5, p.57].

The impact of body image on the course of diabetes has not been considered among numerous factors covered in those guidelines; however, research has shown that body image may be significantly related to both mental and physical health (e.g. [6–9]). Weight dissatisfaction, regardless of body mass index (BMI), is a potentially important psychophysiologic modifier of relationships between BMI and risk of type 2 diabetes [10]. In a study by Carroll et al. [11], conducted in a large sample of 125 people with type 2 diabetes, a relevant correlation between body dissatisfaction and perceived blood glucose control was identified. In addition, participants (especially females) had a higher level of body dissatisfaction, which was associated with discrepancy between current and desired body perceptions (e.g. [12–14]). It is important to pay attention to the cultural context. For example, among people of Latino and African American origins, a very slim body often preferred in Europe is considered unattractive, and having the perfect body means being slightly larger in size, which is not marked by negative emotions [15, 16]. Research into body image and related constructs (e.g., weight-related self-stigma) has been conducted also in other regions (e.g. [9, 17–19]). However, the majority of available studies are limited to assessing body perception in terms of weight (normal weight/overweight) (e.g. [20]) and its potential negative impact on mental and physical well-being, without exploring its role and implications in the context of diabetes. Of note, research indicates that body image is a much more complex and multifaceted construct than expected [21].

Body image is “a system of beliefs and self-esteem with one’s appearance (cognitive aspect), built on internalized fashion patterns and information from the environment (social aspect), accompanied by specific emotions (affective aspect) and behavior (behavioral aspect)” [22, p.20]. Perceived as an internal mental structure, body image is relatively constant during the life cycle; however, intense and chronic somatic or sociocultural stimuli may contribute to its destabilization and, consequently, change. The affective attitude to the body is particularly susceptible to situational and temporal changes [23]. Imposing frequent control behaviors (e.g. monitoring glycemic levels and body weight changes) on patients without prior preparation and support in adapting to changes in their bodies and image may lead to the escalation of negative internal experiences [24]. This is influenced not only by objective changes in appearance but also, more importantly, by their subjective perception. Under the influence of a chronic illness such as diabetes and its complications, an individual may develop a sense of loss of an important aspect of their integral identity, and the body may be experienced as alien and generate numerous negative emotions, which is also important for the person’s global self-esteem [25]. A negative attitude to one’s body has a number of consequences for human functioning. On one hand, it is a risk factor for mental disorders such as depression or eating disorders [26]. On the other hand, in certain situations, it may contribute to the adoption or avoidance of healthy behaviors, such as a healthy diet and physical activity [27], which provide the basis for the treatment of diabetes.

One of the tools for body image assessment, which excels in its multidimensionality, is the Body Esteem Scale (BES); [28]. It has been used in research in different cultures and regions (e.g. among Hmong and Caucasian Americans [29], Spanish population [30], in China, South Korea, and the United States [31], Japan [32], Poland [33], United States, Australia, United Kingdom, Canada, New Zealand, Austria, South Africa, Greece, Vietnam and the Philippines [34]), including studies on large cross-cultural populations [35–37]. The dimensions of BES encompass areas relevant to functioning of people with diabetes, as mentioned in the ADA guidelines [5]. The tool has also a different structure for males and females, thus taking into account gender differences in body perception, often highlighted in the literature (e.g. [38]).

The purpose of this study was to compare the body self-esteem of adults with type 2 diabetes with norms for the general Polish population and to investigate the relationship between body self-esteem and the psychological and clinical characteristics of the course of diabetes. Due to the fact that, at the very beginning of diagnosis and treatment (as a part of psychoeducation), patients are informed that the main mechanism of the disease is the lack of response of their body to its own insulin and that obesity is a factor that significantly increases the risk of developing diabetes [5], we expected that the body image of people with diabetes will be significantly deviated from the norms for the general population. Since the negative body image is associated with poorer emotional condition, which seems important for diabetes management, we also hypothesized that there would be relationships between the negative body image and variables that reflect functioning in diabetes. We also predicted that—in line with the BES tool structure, which is different for men and women—we would observe differences between men and women in terms of body image. Owing to the sociocultural context, which still (even if less markedly than several years ago) seems to be more burdensome for women in terms of visual expectations, we also hypothesized that relationships between women’s body image and functioning in various areas would be stronger.

## Materials and methods

### Participants

The study included a subsample of 135 adult patients of the Diabetes Outpatient Clinic of the Mazovian Bródnowski Hospital in Warsaw, Poland, treated for type 2 diabetes, who participated in the International Prevalence and Treatment of Diabetes and Depression (INTERPRET-DD) study [39]. One hundred individuals (49 women and 51 men) aged 35 to 66 years (M = 58.37; SD = 7.67 and M = 57.55; SD = 7.95, respectively) undergoing treatment agreed to take part in this study (see Table 1). Nineteen persons refused to participate, and 16 individuals did not complete the set of questionnaires. Participants with missing data did not differ in terms of age from those with complete data (*t*(56.32) = -0.30; *P* = .767). There were also no differences in sex distribution (χ^2^ = 0.11; *P* = .738). All participants provided written informed consent before taking part in the study.

**Table 1:** Participant characteristics.

Variables	Women			Men			*t* test		
	*M*	*SD*	Min.–max.	*M*	*SD*	Min.–max.	*t*	*df*	*P*
Age (years)	58.37	7.67	38–66	57.55	7.95	35–66	0.52	98	.602
Diabetes duration (years)	9.22	7.26	1–31	11.25	7.13	1–37	-1.41	98	.161
Hamilton Rating Scale for Depression	8.37	7.26	0–29	6.46	7.49	0–27	1.29	97	.201
Well-Being Index	14.22	6.89	0–25	13.90	7.10	0–25	0.23	98	.818
Body mass index	30.07	5.26	18.44–40.96	30.70	5.50	20.52–44.98	-0.58	98	.563
Glycated hemoglobin (%)	6.97	1.08	4.40–9.00	7.33	1.50	4.99–11.30	-1.35	97	.180
Problem Areas in Diabetes Scale	19.45	19.74	0–62	14.06	15.31	0–57	1.53	98	.129

### Procedure

The study design was approved by the Bioethical Committee at Medical University of Warsaw (approval number KB/21/A/2017), and the study took place in the Diabetes Outpatient Clinic at the Mazovian Bródnowski Hospital in Warsaw, Poland, which was one of the centers participating in the INTERPRET-DD study (see [39]). Study patients were recruited consecutively (all those visiting a diabetologist, at any hour, on 2 days of the week). Following consultation with a diabetologist, enrolled patients with type 2 diabetes were informed about the ongoing study, and, upon providing informed consent, completed a set of questionnaires and underwent a psychiatric examination including Hamilton Rating Scale for Depression assessment conducted by trained psychiatrists, in line with the INTERPRET-DD protocol described in detail in an article by Lloyd et al. [39]. In Warsaw, they additionally completed BES [28]. The exclusion criteria were as follows: being diagnosed with type 2 diabetes for <12 months, which is a typical period of adaptation to this diagnosis; inability to communicate or cognitive impairment precluding completion of the questionnaires; and life-threatening comorbidities such as cancer or stroke in the previous 6 months. BMI and HbA1c data were taken from medical records of the patients.

### Materials

Study participants completed BES [28] translated by Małgorzata Lipowska and Mariusz Lipowski, who also elaborated norms for the Polish population depending on age ranges [21]. The set included 35 statements on body parts and their functions. Levels of satisfaction with individual body dimensions were recorded on a 5-point Likert scale, where 1 denotes a definitely negative emotional attitude, 3—a neutral attitude, and 5—a definitely positive attitude. Depending on the participant’s gender, 3 subscales describing body image were identified. For women those included:

1. The Sexual Attractiveness scale that examined the emotional attitude to those aspects of one’s appearance that may only be modified by cosmetic procedures or plastic surgeries—the areas of the face (eyes, nose, mouth, chin), chest/bust, cheeks/bone, sexual organs—and sex drive, sexual activity, body hair, and body odor.
2. The Weight Concern scale comprising the emotional attitude to those body dimensions that may be modified through exercise and diet. Those included appetite, thighs, waist, buttocks, body structure, hips, legs, abdomen, weight, and body shape.
3. The Physical Condition scale that focused on the emotional attitude to overall physical fitness, strength, and health. It involved body parts and functions such as reflex, physical endurance, energy level, muscle strength, biceps, motor coordination, agility, health, and fitness.

In contrast, in men, body image was assessed with:

1. The Physical Attractiveness scale that referred to the evaluation of those features that in general greatly affect the perception of a man as handsome, including facial features as well as body parts such as hips or feet. The sexual element played a minor role in the overall perception of the physical attractiveness of men.
2. The Upper Body Strength scale that consisted of not only the assessment of individual body parts (e.g. chest or biceps) but also their functions and one’s skills, which provide a basis for the assessment of human strength and activity.
3. The Physical Condition scale that assessed the perception of body endurance, strength, and agility. It examined the emotional attitude to overall physical fitness, health, and strength. This included such aspects as weight, motor coordination, appetite, reflexes, physical endurance, health, agility, physical condition, energy level, body shape, and abdomen.

The original version of BES has a generally good test-retest reliability at a 3-month interval (*r* = 0.75–0.87, except the physical attractiveness subscale in men where *r* = 0.58). In the Polish version, the reliability of subscales is adequate for both females (α ranged from 0.80 to 0.89) and males (α ranged from 0.85 to 0.88). Similarly, the reliability of the entire tool is α = 0.93 (0.94 for men and 0.92 for women). The tool has established Polish norms and may be incorporated in diagnostic tests. The standardization group consisted of 4298 participants at different levels of education, of whom 1865 were women aged 16 to 80 years (M = 29.92; SD = 12.85), and 2433 were men aged 16 to 78 years (M = 28.74; SD = 11.50) [21].

Another questionnaire completed by study participants was the World Health Organization-Five Well-Being Index (WHO-5; [40]). It is a tool that measures subjective well-being and consists of 5 items assessed on a 6-point Likert scale. The original English version has good psychometric properties [41]. Confirmatory factor analysis showed a single-factor structure of both the English version [42] and its Polish translation [43]. The Polish version has satisfactory reliability (α = 0.87) and good convergent validity (*r* = -0.75, *P* <0.001 for the Problem Areas in Diabetes (PAID); [44] scale and *r* = 0.52, *P* <0.001 for the Patient Health Questionnaire [43, 45, 46]. The raw score ranges between 0 (the worst possible well-being) and 25 (the best possible well-being), and a score below 13 indicates poor well-being [43].

The 17-item version of the Hamilton Rating Scale for Depression (HAM-D); [47] is an objectified tool for the assessment of depressive symptoms by a trained clinician following the Interview Guide for the Hamilton Depression Rating Scale (SIGH-D); [48]. The inter-rater reliability (*r* = 0.94, *P* <0.001) and correlation between the HAM-D score and a psychiatrist’s global rating (*r* = 0.89) are high [49]. The psychometric properties of the Polish version have not been analyzed to date, but the tool is widely used in clinical trials. The suggested ranges of interpretation are: 0 to 7 points—normal condition, 8 to 16 points—mild depression, 17 to 23 points—moderate depression, and 24 points or more—severe depression [50].

The PAID [51] scale has proven psychometric properties and is used worldwide to assess diabetes-related emotional distress [44, 52] both in people with type 1 diabetes and those with type 2 diabetes [53]. The score equal to or greater than 40 suggests the presence of severe diabetes-specific emotional problems [54]. The English version of PAID comprises 4 subscales that describe the severity of problems related to negative emotions, treatment, food, and lack of social support. According to recent research, the Polish version does not allow one to distinguish the subscales, although the whole scale is reliable and accurate [43].

BMI and HbA_1c_ levels were also relevant indicators of diabetes-related health status. The reference BMI for adults ranges between 18.5 and 24.9. Values below this range suggest underweight, while exceeding the maximum value indicates overweight. A value of 30 or greater indicates obesity [55]. In patients with diabetes, the recommended value of blood glucose level as expressed by HbA_1c_ is no more than 7% (53 mmol/mol) [56].

### Statistical analyses

Owing to the normal distribution of data on body image dimensions, the Student *t* test was performed to compare the body image of the study participants with type 2 diabetes with standardized data from the Polish BES. If the assumption of equal variances was not met, the Welch t test was performed. Then, the Hedges *g* was calculated, which provided a measure of effect size weighted according to the relative size of each sample. This measure of effect size is adequate in cases where sample sizes differ from each other. The Hedges *g* values were interpreted as recommended by Cohen [57]. Thus, the following rule of thumb was used: 0.2—small effect; 0.5—medium effect; 0.8—large effect.

The presence of relationships between body image variables and psychological, behavioral, and health-related indicators was verified by correlation analysis based on the Pearson correlation coefficient or Spearman rank correlation method; the Spearman rank order correlation coefficient was used to test the strength and direction of the association between body image dimensions and variables with non-normal data distribution (WHO-5, HAM-D, PAID, HbA_1c_ [in men]). The strength of relationships between variables was assessed according to the following criteria [58]: 0.90 to 1.00 (-0.90 to -1.00)—very strong; 0.70 to 0.90 (-0.70 to -0.90)—strong; 0.50 to 0.70 (-0.50 to -0.70)—moderate; 0.30 to 0.50 (-0.30 to -0.50)—weak; .00 to .30 (.00 to -.30)—negligible.

In the next step, multiple regression analyses were conducted to assess the effect of each body image dimension on well-being (WHO-5), depression (HAM-D), BMI, HbA_1c_, and emotional distress (PAID), while controlling for other dimensions. We employed the bootstrap method with bias-corrected and accelerated (BCa) bootstrap confidence intervals (CIs). To calculate sufficiently accurate 95% BCa CIs, 1000 bootstrapped samples were used [59].

The variance inflation factors (VIFs) were estimated in order to detect possible multicollinearity between predictors in multiple regression analysis. A value of VIF greater than 10 indicated a strong correlation with other predictors [60, 61]. However, more restrictive criteria have been recommended recently [61], whereby a VIF value greater than 5 indicates potential multicollinearity. To test the assumption of independent errors, the Durbin–Watson test was conducted. The obtained value less than 1 or greater than 3 indicated the presence of autocorrelation among residuals [59].

The statistical analyses were carried out using the SPSS software, version 27, for Windows. Statistical significance for all conducted analyses was established at *P* <0.05.

## Results

### Participant characteristics

Study participants were aged 35 and 66 years and had a history of diabetes lasting for 1 to 37 years. Mean scores of measures of depression (HAM-D), well-being (WHO-5), and diabetes-related distress (PAID) were within reference ranges. There were no differences between men and women in terms of age and diabetes duration (see Table 1). The mean scores in the 17-item version of the HAM-D may suggest slightly decreased mood in women compared with men; however, this difference was nonsignificant. Both women and men had average BMI values slightly above the threshold of overweight and class 1 obesity. The mean level of HbA_1c_ in comparison to values recommended by the Polish Diabetes Association (7%) [56] was at the reference threshold in women and slightly above that in men. None of the differences between genders was significant. Detailed results are presented in Table 1.

### Comparison of the body image of individuals with type 2 diabetes with the Polish norms

In comparison to women from the general Polish population, females with type 2 diabetes obtained significantly poorer scores on the Physical Condition subscale according to the Welch *t* test (*t*(49.6286) = -2.20; *P* = 0.033). The effect size for this analysis (*Hedges g* = 0.39) exceeded the Cohen convention [57] for a small effect. Men with type 2 diabetes had significantly lower scores for Upper Body Strength (*t*(2482.00) = -2.18; *P* = 0.030) compared with men from the general Polish population. The effect size in this dimension was small (Hedges *g* = 0.31). Men with type 2 diabetes had also significantly poorer scores on the Physical Condition subscale (*t*(2482.00) = -4.90; *P* <0.001) than those from the general population, with a medium effect size (Hedges *g* = 0.69). No significant differences were found for other dimensions. Detailed results are presented in Table 2.

**Table 2:** Comparison of the study participants’ body image and norms for the general Polish population.

BES subscale	Study sample				General population^a^			*t* test		
	N	*M*	*SD*	Min.–max.	N	*M*	*SD*	*t*	*df*	*P*
Women										
*Sexual Attract*.	48	48.58	8.88	30–64	1865	48.86	7.05	-0.22^b^	48.5370	0.829
*Weight Concern*	49	31.20	8.36	16–49	1865	32.64	8.45	-1.18	1912	0.239
*Physical Condition*	49	30.71	7.11	16–45	1865	32.96	5.69	-2.20 ^b^	49.6286	0.033
Men										
*Physical Attract*.	49	40.21	6.31	30–55	2433	40.48	6.56	-0.29	2480.00	0.775
*Upper Body Strength*	51	32.16	6.60	16–44	2433	33.97	5.86	-2.18	2482.00	0.030
*Physical Condition*	51	42.44	9.70	18–61	2433	48.30	8.42	-4.90	2482.00	<0.001

*Note*: BES–Body Esteem Scale; Attract.–Attractiveness

^a^ Means and standard deviations for the general population were derived from “Polish normalization of the Body Esteem Scale” by Lipowska, M. Lipowski M., [21]

^b^ The Welch *t* test was used, as the assumption of equal variances was not met.

### Correlations between the body image and the participants`functioning in the studied areas

The WHO-5, HAM-D, and PAID scores showed non-normal distribution both in women and men, and data on HbA_1c_ did not meet the normality assumption in men. Thus, the Spearman rank correlation method was used to investigate relationships between body image dimensions and well-being, depression, emotional distress, and HbA_1c_ levels (in men). Detailed results of correlational analysis are presented in Table 3.

**Table 3:** Results of the correlational analysis of body image and different areas of functioning.

BES	WHO-5		HAM-D		BMI		HbA_1c_		PAID	
**Women**	***r***_***s***_	***P***	***r***_***s***_	***P***	***r***	***P***	***r***	***P***	***r***_***s***_	***P***
*Overall result*	.427	.002	-.430	.002	-.239	.101	-.386	.007	-.452	.001
*Sexual Attract*.	.339	.018	-.411	.004	-.160	.277	-.392	.006	-.329	.023
*Weight Concern*	.259	.073	-.220	.128	-.362	.010	-.333	.019	-.330	.021
*Physical Condition*	.490	< .001	-.473	.001	-.026	.862	-.299	.037	-.587	< .001
**Men**	***r***_***s***_	***P***	***r***_***s***_	***P***	***r***	***P***	***r***_***s***_	***P***	***r***_***s***_	***P***
*Overall result*	.490	< .001	-.087	.566	-.445	.002	.118	.433	-.334	.022
*Physical Attract*.	.317	.026	.055	.709	-.253	.080	.089	.549	-.214	.141
*Upper Body Strength*	.487	< .001	-.152	.293	-.338	.015	.134	.354	-.372	.007
*Physical Condition*	.547	< .001	-.193	.183	-.531	< .001	.006	.965	-.378	.007

*Note*. BES–Body Esteem Scale; WHO-5 –World Health Organization-Five Well-Being Index; HAM-D–Hamilton Rating Scale for Depression; BMI–body mass index; HbA_1c_ –glycated hemoglobin; PAID–Problem Areas in Diabetes; Attract.–Attractiveness

#### Body image and subjective well-being

In women, subjective well-being (WHO-5) was positively and moderately correlated with total BES scores (*r*_*s*_ = 0.43; *P* = 0.002) and the Physical Condition subscale (*r*_*s*_ = 0.49; *P* <0.001). There was also a positive, weak correlation between well-being and Sexual Attractiveness (*r*_*s*_ = 0.34; *P* = 0.018). In men, subjective well-being was positively and moderately correlated with both the overall BES score (*r*_*s*_ = 0.49; *P* <0.001) and its individual dimensions—Physical Condition (*r*_*s*_ = 0.55; *P* <0.001) and Upper Body Strength (*r*_*s*_ = 0.49; *P* <0.001). The correlation between well-being and the Physical Attractiveness subscale was positive and weak (*r* = 0.32; *P* = 0.026).

#### Body image and severity of depressive symptoms

In women, the severity of depressive symptoms was negatively and moderately correlated with the overall BES score (*r*_*s*_ = -0.43; *P =* 0.002) and its dimensions—Sexual Attractiveness (*r*_*s*_ = -0.41; *P* = 0.004) and Physical Condition (*r* = -0.47; *P* = 0.001). Concerning men, no significant correlations were found between depression and body image variables.

#### Body image and body mass index

In women, BMI was negatively and weakly correlated with the BES Weight Concern (*r* = -0.36; *P* = 0.010) dimension. In men, BMI was negatively and moderately correlated with the overall BES score (*r* = -0.45; *P* = 0.002) and Physical Condition (*r* = -0.53; *P* <0.001). There was also a negative and weak correlation between BMI and Upper Body Strength (*r* = -0.34; *P* = 0.015).

#### Body image and glycated hemoglobin levels

In women, the HbA_1c_ level was negatively and weakly correlated with the overall BES score (*r* = -0.39; *P* = 0.007) and all of its dimensions: Sexual Attractiveness (*r* = -0.39; *P* = 0.006), Weight Concern (*r* = -0.33; *P* = 0.019), and Physical Condition (*r* = -0.30; *P* = 0.037). In men, however, no significant correlations were noted between the aforementioned variables.

#### Body image and diabetes-related distress

In women, diabetes-related distress (PAID score) was negatively and moderately correlated with the overall BES score (*r*_*s*_ = -0.45; *P* = 0.001) and Physical Condition (*r*_*s*_ = -0.59; *P* <0.001), and a weak correlation was found for Sexual Attractiveness (*r*_*s*_ = -0.33; *P* = 0.023) and Weight Concern (*r*_*s*_ = -0.33; *P* = 0.021). In men, diabetes-related distress was negatively and weakly correlated with the overall BES (*r*_*s*_ = -0.33; *P* = 0.022) and its dimensions—Upper Body Strength (*r*_*s*_ = -0.37; *P* = 0.007) and Physical Condition (*r*_*s*_ = -0.38; *P* = 0.007).

### Results of regression analysis

#### Body image dimensions as predictors of functioning in different areas in women with type 2 diabetes

In women, the VIF values were between 1.99 and 2.05, which indicated no multicollinearity (see Table 4). The Durbin–Watson statistics for those data ranged from 1.73 to 2.46. Thus, there were no issues related to multicollinearity and autocorrelations in residuals.

**Table 4:** Results of multiple regression analysis for body image dimensions predicting functioning in different areas among women with type 2 diabetes.

**DV: WHO-5**													
**Predictors**	*B*	*SE*			β			*t*		95% BCa CI			VIF
Sexual Attractiveness	0.281	0.508			0.090			0.491		-0.655; 1.481			1.985
Weight Concern	-0.060	0.554			-0.018			-0.103		-1.038; 0.993			1.822
Physical Condition	1.791	0.832			0.457			2.462		0.043; 3.465			2.045
Model summary: Durbin–Watson value = 2.46; R^2^ = .26; R^2^_adj_ = .21; F(3, 44) = 5.13; *P* = .004													
**DV: HAM-D**	*B*		*SE*			β			*t*			95% BCa CI	
Sexual Attractiveness	-0.110		0.135			-0.133			-0.725			-0.400; 0.118	
Weight Concern	0.117		0.120			0.135			0.764			-0.117; 0.381	
Physical Condition	-0.494		0.198			-0.481			-2.575			-0.888; -0.069	
Model summary: Durbin–Watson value = 2.22; R^2^ = .25; R^2^_adj_ = .20; F(3, 44) = 4.88; *P* = .005													
**DV: BMI**													
Sexual Attractiveness	-0.037			0.106			-0.061		-0.320			-0.239; 0.157	
Weight Concern	-0.337			0.100			-0.533		-2.912			-0.498; -0.111	
Physical Condition	0.253			0.150			0.338		1.744			-0.020; 0.530	
Model summary: Durbin–Watson value = 1.85; R^2^ = .19; R^2^_adj_ = .14; F(3, 44) = 3.46; *P* = .024													
**DV: HbA**_**1c**_ **(%)**													
Sexual Attractiveness	-0.032			0.022			-0.264		-1.365			-0.068; 0.013	
Weight Concern	-0.012			0.024			-0.091		-0.494			-0.066; 0.031	
Physical Condition	-0.017			0.029			-0.110		-0.561			-0.095; 0.051	
Model summary: Durbin–Watson value = 2.26; R^2^ = .17; R^2^_adj_ = .11; F(3, 44) = 3.01; *P* = .040													
**DV: PAID**													
Sexual attractiveness	0.024			0.436			0.011		0.059			-0.777; 0.915	
Weight concern	0.173			0.271			0.073		0.429			-0.342; 0.779	
Physical condition	-1.652			0.544			-0.593		-3.278			-2.732; -0.657	
Model summary: Durbin–Watson value = 1.73; R^2^ = .30; R^2^_adj_ = .25; F(3, 44) = 6.17; *P* = .001													

*Note*: BCa CI– 95% bias-corrected and accelerated confidence intervals. WHO-5 –World Health Organization-Five Well-Being Index; HAM-D–Hamilton Rating Scale for Depression; BMI–body mass index; HbA_1c_ –glycated hemoglobin; PAID–Problem Areas in Diabetes;

The multiple regression analysis concerning well-being (WHO-5) in women with type 2 diabetes led to the construction of a model that explained 21% variation in WHO-5 results (R^2^_adj_ = .25; F(3, 44) = 5.13; *P* = .004). The model for depression severity (HAM-D) accounted for 20% variation in HAM-D scores (R^2^_adj_ = .20; F(3, 44) = 4.88; *P* = .005), whereas the model including PAID as an outcome variable, for 25% variation in diabetes-related emotional distress (R^2^_adj_ = .25; F(3, 44) = 6.17; *P* = .001). The models including BMI and HbA_1c_ as DVs explained their 14% and 11% variations, respectively (see Table 4).

As presented in Table 4, Physical Condition was a significant predictor of well-being, depression, and diabetes-related distress. The higher the Physical Condition score, the better was the well-being (B = 1.79; 95% BCa CI: 0.04–3.47), the less severe was the depression (B = -0.49; 95% BCa CI: -0.89 to -0.07), and the less severe was the diabetes-related emotional distress (B = -1.65; 95% BCa CI: -2.73 to -0.66).

Weight Concern was a significant predictor in the model related to BMI. The lower the Weight Concern score, the greater was the BMI level (β = -0.34; 95% BCa CI: -0.50 to -0.11).

#### Body image dimensions as predictors of functioning in different areas among men with type 2 diabetes

The VIF values in men ranged from 3.25 to 3.76, which indicated that the assumption of no multicollinearity was met (see Table 5). The Durbin–Watson statistics ranged from 1.88 to 2.01. Thus, there was no issue with autocorrelations in residuals.

**Table 5:** Results of multiple regression analysis for body image dimensions predicting functioning in different areas among men with type 2 diabetes.

**DV: WHO-5**													
**Predictors**	*B*	*SE*			β			*t*		95% BCa CI			VIF
Physical Attractiveness	-0.973	1.013			-0.218			-0.968		-3.066; 0.516			3.251
Upper Body Strength	0.377	1.015			0.089			0.370		-1.278; 2.330			3.739
Physical Condition	1.887	0.678			0.648			2.679		0.534; 3.490			3.763
Model summary: Durbin–Watson value = 2.01; R^2^ = .32; R^2^_adj_ = .27; F(3, 44) = 6.76; *P* < .001													
**DV: HAM-D**	*B*		*SE*			β			*t*			95% BCa CI	
Physical Attractiveness	0.261		0.277			0.223			0.889			-0.305; 0.867	
Upper Body Strength	0.023		0.276			0.021			0.079			-0.574; 0.495	
Physical Condition	-0.482		0.171			-0.616			-2.335			-0.806; -0.093	
Model summary: Durbin–Watson value = 1.88; R^2^ = .20; R^2^_adj_ = .14; F(3, 43) = 3.49; *P* = .024													
**DV: BMI**													
Physical Attractiveness	0.206			0.166			0.241		1.110			-0.151; 0.559	
Upper Body Strength	0.204			0.182			0.253		1.086			-0.146; 0.618	
Physical Condition	-0.527			0.128			-0.949		-4.055			-0.792; -0.300	
Model summary: Durbin–Watson value = 2.36; R^2^ = .36; R^2^_adj_ = .32; F(3, 44) = 8.22; *P* < .001													
**DV: HbA**_**1c**_ **(%)**													
Physical Attractiveness	0.019			0.058			0.076		0.287			-0.113; 0.122	
Upper Body Strength	0.078			0.061			0.344		1.207			-0.043; 0.204	
Physical Condition	-0.060			0.041			-0.385		-1.343			-0.140; 0.034	
Model summary: Durbin–Watson value = 1.97; R^2^ = .05; R^2^_adj_ = -.02; F(3, 44) = 0.75; *P* = .530													
**DV: PAID**													
Physical Attractiveness	0.405			0.794			0.163		0.650			-1.299; 2.116	
Upper Body Strength	-0.025			0.731			-0.011		-0.040			-1.486; 1.568	
Physical Condition	-0.792			0.454			-0.491		-1.816			-1.586; -0.035	
Model summary: Durbin–Watson value = 1.95; R^2^ = .15; R^2^_adj_ = .09; F(3, 44) = 2.51; *P* = .071													

*Note*: BCa CI– 95% bias-corrected and accelerated confidence intervals. WHO-5 –World Health Organization-Five Well-Being Index; HAM-D–Hamilton Rating Scale for Depression; BMI–body mass index; HbA_1c_ –glycated hemoglobin; PAID–Problem Areas in Diabetes;

Among men with type 2 diabetes, the multiple regression model for well-being (WHO-5) explained 27% variation in WHO-5 results (R^2^_adj_ = .27; F(3, 44) = 6.76; *P* < .001). The model for depression severity explained 14% variation in HAM-D scores (R^2^_adj_ = .14; F(3, 43) = 3.49; *P* = .024), and the model for BMI as an outcome variable explained 32% variation (R^2^_adj_ = .32; F(3, 44) = 8.22; *P* < .001). The models including HbA_1c_ and PAID as DVs yielded nonsignificant results (see Table 5), although Physical Condition appeared to be a significant predictor of diabetes-related emotional distress (B = -0.79; BCa CI: -1.59 to -0.04), and the lower the Physical Condition score, the higher were the PAID results.

As presented in Table 5, Physical Condition was the only significant predictor of well-being, depression, and BMI among men. The higher the Physical Condition score, the better was the well-being (B = 1.89; 95% BCa CI: 0.53–3.49), the less severe was the depression (B = -0.48; 95% BCa CI: -0.81 to -0.09), and the lower was the BMI (B = -0.53; 95% BCa CI: -0.79 to -0.30).

## Discussion

To our best knowledge, this is the first study that both compares the detailed body self-esteem of adults with type 2 diabetes with norms for the general population and investigates the relationship between body self-esteem and the psychological and clinical characteristics of the course of diabetes. With a low refusal rate, the study sample was relatively representative for the population of patients with type 2 diabetes treated in a good-quality outpatient diabetes clinic in Poland. Our results confirmed the hypotheses and demonstrated that the body image of adult men and women with type 2 diabetes is significantly poorer than in the general population. We also showed the important role of gender for both body image and its relationships with mental and physical health.

As concluded by Bays et al. [62], individuals with type 2 diabetes may differ from those without diabetes in terms of body image perception. Indeed, in our study, both males and females with type 2 diabetes exhibited significant differences in body image perception as compared with the available norms, including lower Physical Condition scores and, in male patients, also lower Upper Body Strength.

From the clinical point of view, the most interesting finding is a moderate correlation between total BES results and HbA_1c_ levels of -.386. It is very high in comparison to correlations found between HbA_1c_ and other psychosocial factors, which are usually below 0.2 (e.g. [63–66]). In this context, a correlation of 0.118 among men also has some significance. If those findings are confirmed in larger, multicenter studies and prospective studies, body image, at least in women, can be considered a very important psychological factor influencing glucose level and eventually the course of diabetes, including the risk of complications.

Our study provided further findings improving the understanding of problems related to body image. Another significant relationship was observed in women, but not in men, between a positive body image (both in the overall BES score and all its dimensions: Sexual Attractiveness, Weight Concern, and Physical Condition) and a low level of HbA_1c_. This corresponds to the results of a study indicating that perceived body image (appearance evaluation) is positively correlated with self-reported health behavior among patients with diabetes with a BMI greater than 24 [67]. The relationship between body dissatisfaction and perceived blood glucose control in women was identified also by Carroll et al. [11].

A positive body image proved to be significantly correlated with overall subjective well-being (as measured by WHO-5). In women, this was additionally noted in Physical Condition and Sexual Attractiveness dimensions; in men, in Physical Attractiveness, Upper Body Strength, and Physical Condition subscales. This may be due to the fact that body image is one of the components of overall self-esteem [68], which in turn influences one’s mood. In women, a more positive body image was also associated with less severe depressive symptoms. That was observed both in the overall BES score and in Sexual Attractiveness and Physical Condition dimensions. This finding is consistent with results obtained by Carroll et al. [11], who noticed that body dissatisfaction in women with diabetes tends to be linked to more severe depressive symptoms. This might suggest that a positive attitude towards one’s body is conducive to maintaining mental health in individuals with a chronic illness. Negative attitudes under prolonged stress conditions may lead to the escalation of negative internal experiences [24]. This hypothesis, however, would require verification in further studies, especially because no significant correlations were found between depression and body image variables in men. This is in line with our hypotheses about the greater importance of the body image construct among women, and also in line with the results of a study by Furnham, Badmin, and Sneade [69], conducted in a sample of 235 adolescents, which demonstrated that body dissatisfaction affects the concept of self-esteem in girls but not in boys, which highlights gender differences.

Our study also revealed a relationship between a positive body image and less severe diabetes-related distress. In women, significant results were obtained for the overall BES score as well as Physical Condition, Sexual Attractiveness, and Weight Concern dimensions, and in men, for the overall BES score as well as Upper Body Strength and Physical Condition dimensions. When it comes to the clinical characteristics of the course of diabetes, a lower BMI was significantly correlated with a more positive body image both in women (in the Weight Concern dimension) and men (in the overall BES score as well as Physical Condition and Upper Body Strength dimensions).

In order to better understand the observed relationships, we decided to conduct an additional regression analysis. It showed that, indeed, in women, higher Physical Condition scores were significant predictors of better well-being, less severe depression, and less severe diabetes-related distress. On the other hand, lower Weight Concern scores appeared to be significant predictors of a higher BMI. In men, higher Physical Condition scores significantly predicted less severe diabetes-related emotional distress, less severe depression, and a lower BMI yet better well-being. What is important, the models based on body esteem dimensions constructed in this study explained variations regarding well-being, depression, and diabetes-related distress better than models presented in other studies.

Of note, the discussed data were collected in a single center in one country. Therefore, this is only a starting point for further research aimed at, among others, confirming and improving the understanding of the reported relationships. Due to adopted study design, it was not possible to verify if the perception of one’s body was a correct judgement of change in the course of diabetes or a distortion of sound judgment caused by depressive symptoms or other psychological factors. This study was also limited by a relatively good glucose in study participants, which may have had an impact on our findings, in addition to the low levels of reported diabetes-related distress. Therefore, it is possible that if this study had been conducted in individuals with worse level of glucose, the observed relationships would have been even stronger and more significant relationships could have been revealed.

Our findings suggest that identifying challenges related to body image, in addition to other psychological factors, may be important to consider when supporting diabetes self-management. For example, according to Shaban [70], the extent of body image–related distress can lead to suboptimal glycemic control and perpetuate the problem. According to a systematic review by Graham et al. [71], the acceptance of one’s own, even difficult, reality and the ability to cope with it are directly reflected in a person’s quality of life and the level of distress they experience and may even contribute to increased motivation to follow recommendations and to a better control of symptoms. Our findings have practical implications for the treatment of diabetes. They suggest that a patient’s body image should be evaluated during the initial diagnosis, and monitored during follow up visits. Behavioral recommendations regarding diet, weight, and exercise should formulated in a way that does not cause additional negative emotions and stress associated with negative body image, e.g. critical remarks on body image should be avoided. Further research is needed to confirm results obtained in this study. If they occur repeatable, it may be advisable to develop a rapid screening test to assess body image in everyday clinical practice.

## Conclusions

In this study, we observed that body image in adults with type 2 diabetes was significantly worse than in the general Polish population, and the observed differences were gender-specific. Moreover, body image was significantly related to HbA_1c_ levels, especially among women, and to subjective well-being and severity of depression symptoms as well as the level of diabetes-related distress and BMI, which are relevant factors in diabetes care.

## Supporting information

S1 Data (CSV)
